# A temporal banding approach for consistent taxonomic ranking above the species level

**DOI:** 10.1038/s41598-017-02477-7

**Published:** 2017-05-23

**Authors:** Ekaphan Kraichak, Ana Crespo, Pradeep K. Divakar, Steven D. Leavitt, H. Thorsten Lumbsch

**Affiliations:** 1 Department of Botany, Kasetsart University, 50 Ngamwongwan Road, Ladyao, Chatuchak, Bangkok 10900 Thailand; 2 Center for Advanced Studies in Tropical Natural Resources, NRU-KU, Kasetsart University, Chatuchak, Bangkok 10900 Thailand; 3 Departamento de Biología Vegetal II, Facultad de Farmacia, Universidad Complutense de Madrid, Madrid, 28040 Spain; 4 Department of Biology and M.L. Bean Life Science Museum, Brigham Young University, 4102 Life Science Building, Provo, UT 84602 USA; 5 Science & Education, Field Museum of Natural History, 1400 S Lake Shore Drive, Chicago, IL 60605 USA

## Abstract

Comparable taxonomic ranks within clades can facilitate more consistent classifications and objective comparisons among taxa. Here we use a temporal approach to identify taxonomic ranks. This is an extension of the temporal banding approach including a Temporal Error Score that finds an objective cut-off for each taxonomic rank using information for the current classification. We illustrate this method using a data set of the lichenized fungal family Parmeliaceae. To assess its performance, we simulated the effect of taxon sampling and compared our method with the other temporal banding method. For our sampled phylogeny, 11 of the 12 included families remained intact and 55 genera were confirmed, whereas 32 genera were lumped and 15 genera were split. Taxon sampling impacted the method at the genus level, whereas yielded only insignificant changes at the family level. The other available temporal approach also gives a similar cutoff point to our method. Our approach to identify taxonomic ranks enables taxonomists to revise and propose classifications on an objective basis, changing ranks of clades only when inconsistent with most taxa in a phylogenetic tree. An R script to find the time point with the minimal temporal error is provided.

## Introduction

Conservative estimates of the number of species on this planet suggest that at least 8.7 million eukaryotes exist^1^, with the fungal kingdom alone forecast to contain several million species^2^. Overall, up to 100 million species are predicted to occupy the planet^3^. Regardless of the exact number, the sheer magnitude of species diversity requires a classification system that allows effective organization and communication of complex patterns of organismal diversity.

Beginning with Aristotle^4, 5^, organisms were classified according to their similarities. Linnaeus subsequently used a hierarchical classification and understood this pre-Darwinian “natural system” as a reflection of a divine plan of creation^6, 7^, as the idea of evolution was mostly alien to the scientists of the time^4, 5^. However, Linnaeus’ hierarchical classification system persisted and is still in use today. Attempts to develop non-hierarchical systems, such as a quinary system or a periodic system, were unsuccessful^8^.

In a phylogenetic context, taxonomic ranks in this hierarchical classification represent clades with a shared evolutionary history. Since members of a clade are derived from a common ancestor, they often share phenotypic traits. This helps explain why only seven years after Darwin’s seminal book, Haeckel could publish a tree of life, which reinterpreted the classification in a phylogenetic framework^9^. Taxonomic ranks in a hierarchical classification serve an important role for communication among biologists, and other disciplines, for comparative ecological, and conservation studies. However, it should be noted that these ranks are inherently arbitrary; hence there is no absolute definition of specific ranks, which partly explains disparities in ranking among classifications. Taxonomic circumscriptions of the same rank at varying phylogenetic scales lead to a number of potential biases when making comparisons among taxa. These discrepancies have been documented at higher-level taxonomic ranks, which have been shown to circumscribe vastly different phylogenetic scales in a number of cases^8, 10–14^. At the genus level, the current binomial system may lead to frequent name changes, thus making communication difficult and potentially adding confusion^15–19^. As a consequence, comparisons using higher-level ranks across different organismal groups are potentially flawed.

To make taxonomic ranks at supraspecific level more consistent and increase meaningful comparability among higher taxonomic ranks, the use of a standardized time of divergence has been suggested as a universal yardstick for the assignment of ranks^8, 11–13, 20–22^. This means that groups of organisms are given the same rank if they originated in the standardized geological time period.

In this study we use a method that employs time-calibrated chronograms to identify upper and lower thresholds for taxonomic ranks based on the temporal banding approach^11, 13^. To this end, we propose an extension of Holt and Jønsson’s Temporal Error Score^20^ to find an objective cut-off for each taxonomic rank, using the information from the current classification. To illustrate the utility of this temporal banding approach, we apply this modified approach to the most comprehensive phylogeny of the hyperdiverse lichen-forming fungal family Parmeliaceae. We briefly describe the temporal approach with Temporal Error Score^20^ and its computational limitations. Finally, we illustrate how the Temporal Error Score to can be used to find the time point with the minimal error to modify the current classification. Through simulation studies we assess the sensitivity of this method to taxon sampling. Further, we provide an R script to find the time point with the minimal temporal error with this proposed method.

## Temporal inconsistency at the same taxonomic ranks

The chronogram used for the demonstration of this method was reconstructed from a multi-locus alignment, containing six markers (ITS, mtSSU, nuLSU, *RPB*-*1*, *Tsr1*, *Mcm7*) from 340 taxa representing 81 currently accepted genera^23, 24^. Due to a number of recent molecular phylogenetic studies on Parmeliaceae, the majority of the taxa are reciprocally monophyletic in this phylogeny^25^. Fifty-two additional taxa from ten related families^26^ were also included to determine the relationships among the families and to study the temporal inconsistency among the taxa of the same taxonomic ranks, particularly at the genus and family levels.

**Figure 1:**
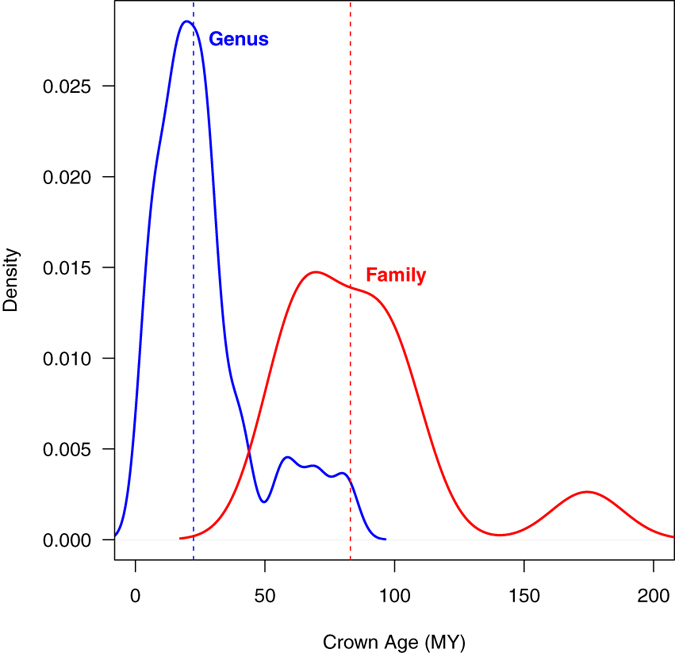
Distribution of crown ages of currently accepted genera (blue) and families (red) in the studied data set (Parmeliaceae, Ascomycota). The median for both ranks indicated by a dashed line.

We used the function getMRCA in the R-package “ape” in order to find the crown age of currently accepted taxonomic groups and found that while the majority of genera and families in this cladogram are about the same age around the median (22 MY for genus and 83 MY for family), a fair number of taxa are far older or younger than the majority of clades (Fig. 1). As has been previously shown in different animal groups^13, 20^, the ranges of taxon ages overlapped between the genus and family ranks, making some of these taxonomic units not comparable even within the same family or order.

## Temporal Error Scores

In an attempt to achieve temporally consistent classification, Holt and Jønsson proposed the Temporal Error Scores as a way to assess the amount of deviation from a single temporal cut-off^20^. The method requires that the tree has been dated (chronogram), and that each tip is assigned to a particular taxonomic group at each rank. This approach first finds the “empirical error” by calculating the difference between the cut-off point and the crown age of each currently accepted taxonomic group and summing it up as an error score. Then, since the empirical error is readily comparable to other scores, the “standardized score” is also calculated by dividing the empirical error with the score computed from random expectations, which are generated from splitting the tree randomly into the same number of monophyletic groups.

While this metric is useful for quantifying the error from the current classification, two issues make it difficult to apply this method with other systems. First, the temporal cut-off points were arbitrarily chosen to generate the roughly same number of monophyletic groups as the current classification, which may not serve as a good criterion, because the current taxonomic groups are also somewhat arbitrary, constantly subjected to either over-lumping or over-splitting. Second, the approach to generate random expectation can be computationally intensive and not readily reproducible. To address these issues, we extend the empirical error score to objectively select the cut-off point without the need to generate the random expectations.

## Minimal Temporal Error Score

Using Holt & Jønsson’s code for taxonomic errors^20^, we applied a series of temporal thresholds from the tip (time = 0) to the root (the tree depth) and calculated an empirical error score at every 1 MY at both the genus and family levels. The temporal threshold with the minimum error score was then used as a cut-off point for the new classification with the temporal approach, in which we recognized all of the monophyletic groups that were more recent than the cut-off as individual taxa at that taxonomic rank. In cases where several time points produced the same error score, the average of those time points was used as a cut-off point. Because of inherently large confidence intervals at each node of the chronogram, we also calculated error margins around the cut-off point to allow some flexibility for reclassification, arbitrarily set to 5%. All of the procedures were performed in the statistical programming R. We also provided the R script to find the time point with minimal error, plot temporal banding on the chronogram, and reclassify based on the new time point on a data depository: http://dx.doi.org/10.5061/dryad.p8n72.

**Figure 2:**
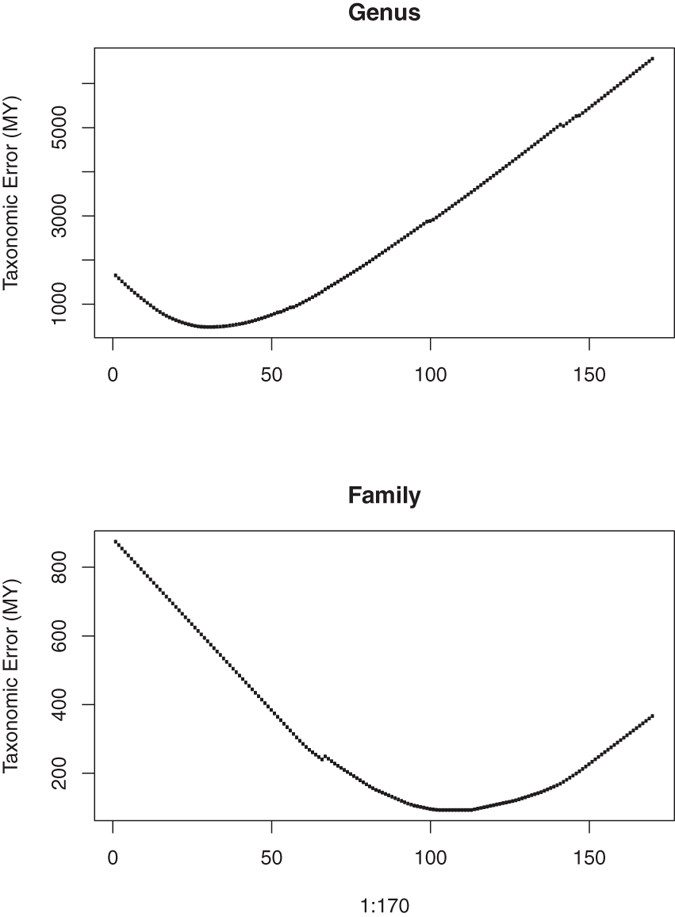
Distribution of empirical error score estimated from the tip (time = 0) to the root (the tree depth) for the genus level (A) and family level (B).

**Figure 3:**
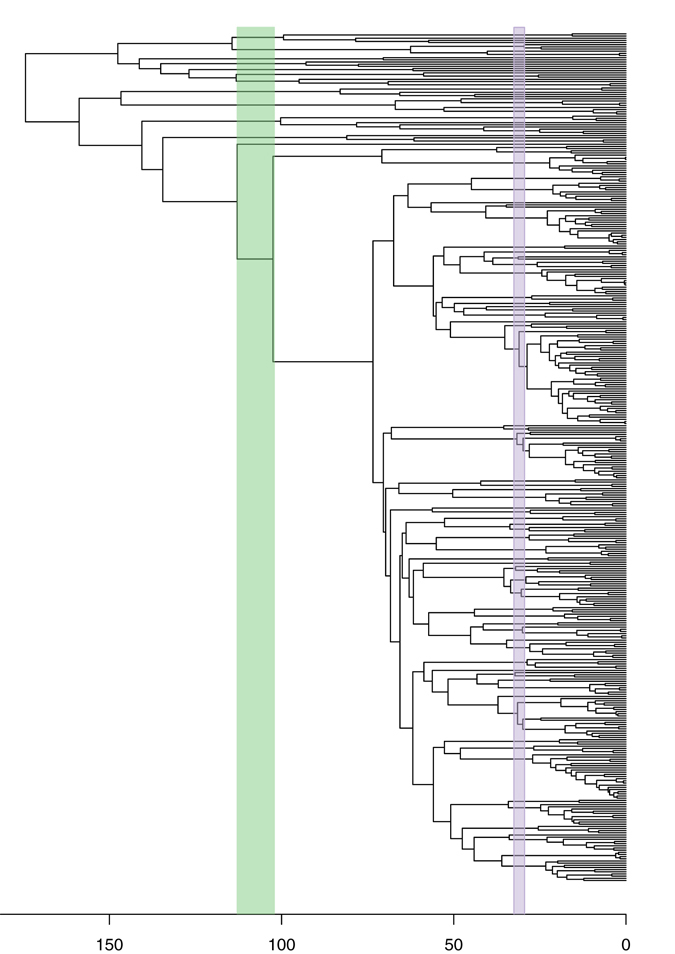
Time-calibrated phylogeny of Parmeliaceae and related families based on a multi-locus data set. Temporal bands for family rank (green) and genus level (pink) indicated.

For our sampled phylogeny, the lowest empirical errors were found at 31 (29.45–32.55) and 107.5 (102.125–112.875) MY for the genus and family, respectively (Fig. 2). We then used the “cutree” function to reclassify the taxonomic groups, based on these time points. With the new cut-off point, 8 families remained intact with 2 families being split. For genera, 42 genera remain the same with 32 genera being lumped and 7 being split (Fig. 3; Supplementary materials). Details of the taxonomic changes are discussed in a companion paper in a mycological journal (manuscript in revision). Instead of using an arbitrary cut-off to maintain the number of taxonomic groups, here we provide a method to maintain the status of most taxonomic groups, while reclassifying the others, using the same age range within the same taxonomic rank.

## Sensitivity to taxon sampling

Taxon sampling poses a challenge to virtually all molecular phylogenetic studies, as it is not always possible to acquire data for every single taxon in a lineage. Since the topology and branch lengths of chronograms have been shown to be sensitive to the amount of taxa being included^27, 28^, temporal methods are likely to be affected by taxon sampling, as they rely heavily on branch lengths. In order to determine the sensitivity of this method to the amount of included taxa, we simulated reduced taxon sampling in our sample tree by randomly removing 10% to 50% of the tips from the tree with the function “drop.tip.” Then, we used our proposed method to find the cut-off point with the minimum lowest empirical error for each of the 500 simulations.

**Figure 4:**
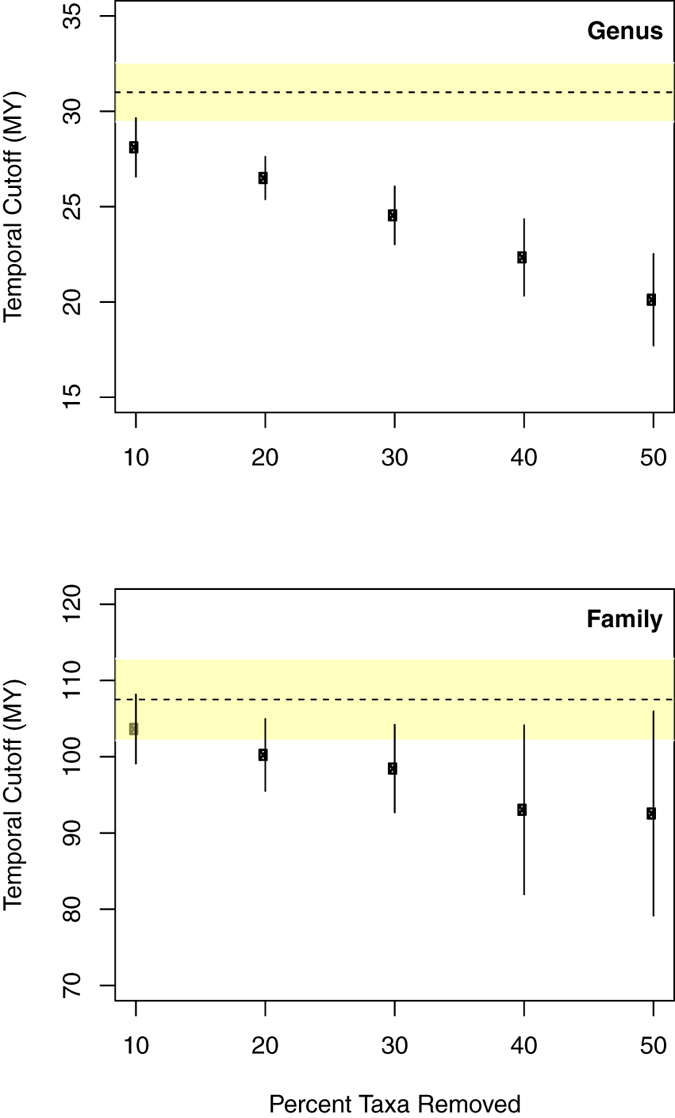
Results of simulations to evaluate sensitivity of temporal banding to taxon sampling. Temporal cut-off indicated as dots (with standard deviation) in relation to amount of removal of taxa from the data set. (A) Temporal cut-off at the genus level. (B) Temporal cut-off at the family level.

The simulation results showed that the cut-off time point changed and got younger as more taxa were removed from the tree (Fig. 4). However, for the family level, the one standard deviation around the average from the simulations still fell within the 5% error margin of the selected time point in the current data. For the genus level, the cut-off points from the simulations were clearly younger than the most comprehensive dataset currently available, even when considering the standard deviation. These results suggest that taxon sampling is critical for the application of this method for temporal classification, but more so at the lower taxonomic ranks, e.g. genus, whereas the higher taxonomic ranks appear less impacted by lower sampling efforts.

## Comparison with the Other Temporal Banding Method

Recently, Jønsson *et al*.^29^ proposed a temporal banding approach that aims to minimize the disruption to the current taxonomy. They developed the metric called “Percent Consistency” which calculates the percentage of the taxa remaining intact after applying a certain cutoff time. This method then chooses the best cutoff time that yields the highest percent consistency. We ran our data through their public available R code and compared the resulting classification and the cutoff time at the genus and family level. The results showed that the cutoff time for the genus level from the Jønsson *et al*. method (28 MY) was close to the lower end of the five-percent band from our method (29.45–32.55 MY), whereas the cutoff times for the family level were nearly identical (107.71 vs. 107.5 MY; Table 1). Our methods generally resulted in more changes in the assignment of genera and families than the Jønsson *et al*.’s method (Table 1). The discrepancy is due to our different metrics for choosing the optimal cutoff point.

**Table 1:** Comparison of resulting new classification from the proposed temporal banding method and the method by Jønsson *et al*.^29^.

	Intact	Lumped	Split	Cutoff Time (MY)
**Genus level**				
Proposed method	55	32	15	31 (29.45–32.55)
Jønsson *et al*.	71	25	5	28.01
**Family level**				
Proposed method	11	0	1	107.5 (102.125–112.875)
Jønsson *et al*.	12	0	0	107.71

The Jønsson *et al*. method^29^ was specifically developed to maintain the current taxonomy, whereas our method relies on the calculation of temporal error scores and aimed more toward having comparable and temporally consistent taxonomic ranks. For many widely studied groups, such as mammals and birds, a temporal banding approach that minimize disruption to current taxonomy might be preferred, because any changes can affect many subsequent uses of the taxonomy. However, in other poorly studied groups, such as bryophytes and fungi, the existing assignment of supraspecific ranks are somewhat arbitrary and subjected to constant changes. In these groups, maintaining the current taxonomy is not a priority. Our method offers an alternative metric to find the temporal cutoff that does not solely focus on maintaining the current supraspecific taxonomic ranks.

## Caveats

The effectiveness of the proposed temporal classification depends on the quality of chronogram reconstruction, which in turns relies on the amount and type of data, alignment methods and taxon sampling. As the minimum empirical error is derived for a particular tree, the absolute time point should not be used across different groups of organisms. For example, the 31-MY cut-off for the genus level cannot readily be applied to any other organismal groups, because it was calculated from the crown ages of the focused groups only.

Monotypic taxa are common across various supraspecific taxonomic ranks and pose a challenge as how to accurately determine the crown age of the taxa with only one tip. The calculation of the temporal error score that we implement here follows the algorithm by Holt and Jønsson^20^, who explicitly state that this method of calculation does not use the crown age of the group and therefore is able to include monotypic taxa in the analysis. However, similar to their work, we did not include monotypic taxa for the crown age distribution analysis.

With different groups of organisms having different evolutionary histories and timelines, trying to find one universal cut-off for each taxonomic rank might not be productive. However, for groups of related organisms, the application of this method on a credible chronogram should allow to objectively find the temporal cut-off for classification of the same rank, while preserving taxonomic status of the majority of the taxa. The method provides an additional tool for erecting new taxa at supraspecific levels in an objective framework, adding to ongoing and growing discussion about temporal banding approaches to taxonomy.

## Electronic supplementary material

Comparison of Current and New Classification
