# Supplementary material for: A temporal banding approach for consistent taxonomic ranking above the species level
Source: Sci Rep. 2017 May 23;7:2297. doi: 10.1038/s41598-017-02477-7 (PMC5442095; doi:10.1038/s41598-017-02477-7)
Supplement: Supplementary file 1 — Comparison of Current and New Classification [file 41598_2017_2477_MOESM1_ESM.pdf]

Supplementary Materials for

A temporal banding approach for consistent taxonomic ranking above the species level

Ekaphan Kraichak, Ana Crespo, Pradeep K. Divakar, Steven D. Leavitt & H. Thorsten Lumbsch

Comparisons between current and new classification according to a temporal approach.

	OldFam	NewFam	OldGen	NewGen
Ahtiana_pallidula	Parmeliaceae	1	Ahtiana	1
Allocetraria_ambigua	Parmeliaceae	1	Allocetraria	1
Allocetraria_flavonigrescens	Parmeliaceae	1	Allocetraria	1
Allocetraria_globulans	Parmeliaceae	1	Allocetraria	1
Allocetraria_stracheyi_AST_02	Parmeliaceae	1	Allocetraria	1
Arctocetraria_andrejewii	Parmeliaceae	1	Arctocetraria	1
Arctocetraria_nigricascens	Parmeliaceae	1	Arctocetraria	1
Cetraria_islandica	Parmeliaceae	1	Cetraria	1
Cetraria_laevigata	Parmeliaceae	1	Cetraria	1
Cetraria_muricata	Parmeliaceae	1	Cetraria	1
Cetraria_nigricans	Parmeliaceae	1	Cetraria	1
Cetraria_odontella	Parmeliaceae	1	Cetraria	1
Cetraria_sepincola_CSE_01	Parmeliaceae	1	Cetraria	1
Cetrariella_delisei	Parmeliaceae	1	Cetrariella	1
Cetrariella_fastigiata	Parmeliaceae	1	Cetrariella	1
Cetrariella_fastigiata_CFA_02	Parmeliaceae	1	Cetrariella	1
Cetreliopsis_rhytidocarpa	Parmeliaceae	1	Cetreliopsis	1
Flavocetraria_cucullata	Parmeliaceae	1	Flavocetraria	1
Flavocetraria_nivalis	Parmeliaceae	1	Flavocetrariella	1
Kaernefeltia_merrillii	Parmeliaceae	1	Kaernefeltia	1
Masonhalea_inermis_551a	Parmeliaceae	1	Masonhalea	1
Masonhalea_richardsonii	Parmeliaceae	1	Masonhalea	1
Melanelia_commixta	Parmeliaceae	1	Melanelicetraria	1
Melanelia_sorediella	Parmeliaceae	1	Melanelicetraria	1
Melanelia_culbersonii_3992	Parmeliaceae	1	Melaneliculbersonia	1
Cetraria_obtusata	Parmeliaceae	1	Neocetraria	1
Nephromopsis_komarovii	Parmeliaceae	1	Nephromopsis	1
Nephromopsis_laureri	Parmeliaceae	1	Nephromopsis	1
Nephromopsis_leucostigma	Parmeliaceae	1	Nephromopsis	1
Nephromopsis_nephromoides	Parmeliaceae	1	Nephromopsis	1
Nephromopsis_pallescens	Parmeliaceae	1	Nephromopsis	1
Tuckermanella_coralligera	Parmeliaceae	1	Tuckermanella	1
Tuckermanella_fendleri	Parmeliaceae	1	Tuckermanella	1
Tuckermannopsis_chlorophylla	Parmeliaceae	1	Tuckermannopsis	1
Tuckermannopsis_ciliaris	Parmeliaceae	1	Tuckermannopsis	1
Tuckermannopsis_orbata	Parmeliaceae	1	Tuckermannopsis	1
Usnocetraria_oakesiana	Parmeliaceae	1	Usnocetraria	1
Vulpicida_canadensis_CAN_15	Parmeliaceae	1	Vulpicida	1
Vulpicida_juniperinus_JUN_07	Parmeliaceae	1	Vulpicida	1
Vulpicida_pinastri	Parmeliaceae	1	Vulpicida	1
Vulpicida_tubulosus_JUN_14	Parmeliaceae	1	Vulpicida	1
Vulpicida_viridis_VIR_10	Parmeliaceae	1	Vulpicida	1
Alectoria_arctica_S146	Parmeliaceae	1	Alectoria	2
Alectoria_nigricans	Parmeliaceae	1	Alectoria	2
Alectoria_ochroleuca	Parmeliaceae	1	Alectoria	2
Alectoria_sarmentosa	Parmeliaceae	1	Alectoria	2
Allantoparmelia_almquistii_5158	Parmeliaceae	1	Allantoparmelia	3
Allantoparmelia_alpicola	Parmeliaceae	1	Allantoparmelia	3
Anzia_colpodes	Parmeliaceae	1	Anzia	4
Anzia_flavotenuis	Parmeliaceae	1	Anzia	5
Anzia_mahaeliyensis	Parmeliaceae	1	Anzia	5
Arctoparmelia_centrifuga	Parmeliaceae	1	Arctoparmelia	6
Arctoparmelia_incurva_MWE10	Parmeliaceae	1	Arctoparmelia	6
Austroparmelina_endoleuca2	Parmeliaceae	1	Austroparmelina	7
Austroparmelina_macrospora	Parmeliaceae	1	Austroparmelina	7
Austroparmelina_pruinata	Parmeliaceae	1	Austroparmelina	7
Austroparmelina_pseudorelicina	Parmeliaceae	1	Austroparmelina	7
Brodoa_atrofusca	Parmeliaceae	1	Brodoa	8

Brodoa_intestiniformis	Parmeliaceae	1	Brodoa	8
Brodoa_oroarctica	Parmeliaceae	1	Brodoa	8
Hypogymnia_bitteri30678	Parmeliaceae	1	Hypogymnia	8
Hypogymnia_hultenii2	Parmeliaceae	1	Hypogymnia	8
Hypogymnia_imshaugii1	Parmeliaceae	1	Hypogymnia	8
Hypogymnia_imshaugii2	Parmeliaceae	1	Hypogymnia	8
Hypogymnia_lophyrea2	Parmeliaceae	1	Hypogymnia	8
Hypogymnia_lugubris3014	Parmeliaceae	1	Hypogymnia	8
Hypogymnia_mollisD79	Parmeliaceae	1	Hypogymnia	8
Hypogymnia_mundata3013	Parmeliaceae	1	Hypogymnia	8
Hypogymnia_physodes	Parmeliaceae	1	Hypogymnia	8
Hypogymnia_pulverata_2277	Parmeliaceae	1	Hypogymnia	8
Hypogymnia_pulverata_2281	Parmeliaceae	1	Hypogymnia	8
Hypogymnia_rugosa3822	Parmeliaceae	1	Hypogymnia	8
Hypogymnia_subphysodes_2280	Parmeliaceae	1	Hypogymnia	8
Hypogymnia_tasmanica3012	Parmeliaceae	1	Hypogymnia	8
Hypogymnia_vittata	Parmeliaceae	1	Hypogymnia	8
Pseudevernia_consocians	Parmeliaceae	1	Pseudevernia	8
Pseudevernia_furfuracea	Parmeliaceae	1	Pseudevernia	8
Bryocaulon_divergens_MWE158	Parmeliaceae	1	Bryocaulon	9
Bryocaulon_pseudosatoanum_YO8239	Parmeliaceae	1	Bryocaulon	9
Bryocaulon_satoanumMWE163	Parmeliaceae	1	Bryocaulon	9
Bryoria_americana_S329	Parmeliaceae	1	Bryoria	10
Bryoria_bicolor_L156	Parmeliaceae	1	Bryoria	10
Bryoria_capillaris	Parmeliaceae	1	Bryoria	10
Bryoria_fremontii	Parmeliaceae	1	Bryoria	10
Bryoria_furcellata_L147	Parmeliaceae	1	Bryoria	10
Bryoria_fuscescens_S56	Parmeliaceae	1	Bryoria	10
Bryoria_glabra_L186	Parmeliaceae	1	Bryoria	10
Bryoria_implexa_L244a	Parmeliaceae	1	Bryoria	10
Bryoria_nadvornikiana_S79	Parmeliaceae	1	Bryoria	10
Bryoria_simplicior_S30b	Parmeliaceae	1	Bryoria	10
Bryoria_smithii_S65	Parmeliaceae	1	Bryoria	10
Bryoria_trichodes	Parmeliaceae	1	Bryoria	10
Bulbothrix_apophysata	Parmeliaceae	1	Bulbothricella	11
Bulbothrix_coronata	Parmeliaceae	1	Bulbothricella	11
Bulbothrix_decurtata	Parmeliaceae	1	Bulbothrix	12
Bulbothrix_isidiza1	Parmeliaceae	1	Bulbothrix	12
Bulbothrix_isidiza2	Parmeliaceae	1	Bulbothrix	12
Bulbothrix_meizospora	Parmeliaceae	1	Bulbothrix	12
Bulbothrix_meizospora2	Parmeliaceae	1	Bulbothrix	12
Bulbothrix_sensibilis	Parmeliaceae	1	Bulbothrix	12
Bulbothrix_setschwanensis	Parmeliaceae	1	Bulbothrix	12
Bulbothrix_tabacina	Parmeliaceae	1	Bulbothrix	12
Canoparmelia_carneopruinata	Parmeliaceae	1	Crespoa	13
Nesolechia_oxyspora_16840	Parmeliaceae	1	Nesolechia	13
Punctelia_aff_bolliana	Parmeliaceae	1	Punctelia	13
Punctelia_borreri2	Parmeliaceae	1	Punctelia	13
Punctelia_pseudocoralloidea	Parmeliaceae	1	Punctelia	13
Punctelia_reddenda	Parmeliaceae	1	Punctelia	13
Punctelia_rudecta	Parmeliaceae	1	Punctelia	13
Punctelia_subflava	Parmeliaceae	1	Punctelia	13
Punctelia_subrudecta3	Parmeliaceae	1	Punctelia	13
Punctelia_ullophylla	Parmeliaceae	1	Punctelia	13
Canoparmelia_caroliniana	Parmeliaceae	1	Canoparmelia	14
Canoparmelia_nairobiensis	Parmeliaceae	1	Canoparmelia	14
Canoparmelia_inhaminensis	Parmeliaceae	1	Crespoa	14
Canoparmelia_schelpei	Parmeliaceae	1	Crespoa	14
Canoparmelia_concrescens	Parmeliaceae	1	Canoparmelia	15

Canoparmelia_texana	Parmeliaceae	1	Canoparmelia	15
Canoparmelia_crozalsiana	Parmeliaceae	1	Crespoa	15
Canoparmelia_Punctelia_sp	Parmeliaceae	1	Newgenus	15
Cetrelia_cetrarioides	Parmeliaceae	1	Cetrelia	16
Cetrelia_olivetorum	Parmeliaceae	1	Cetrelia	16
Cetrelia_pseudolivetorum	Parmeliaceae	1	Cetrelia	16
Coelopogon_abraxus_4253	Parmeliaceae	1	Coelopogon	20
Coelopogon_epiphorellus_4254	Parmeliaceae	1	Coelopogon	20
Coelopogon_epiphorellus_MWE156	Parmeliaceae	1	Coelopogon	20
Cornicularia_normoerica	Parmeliaceae	1	Cornicularia	21
Dactylina_arctica_4855	Parmeliaceae	1	Dactylina	22
Dactylina_ramulosa_4902	Parmeliaceae	1	Dactylina	22
Emodomelanelia_masonii	Parmeliaceae	1	Emodomelanelia	23
Esslingeriana_idahoensis_4823	Parmeliaceae	1	Esslingeriana	24
Melanelia_hepatizon	Parmeliaceae	1	Melanelia	24
Melanelia_stygia	Parmeliaceae	1	Melanelia	24
Evernia_divaricata	Parmeliaceae	1	Evernia	25
Evernia_mesomorpha	Parmeliaceae	1	Evernia	25
Evernia_prunastri	Parmeliaceae	1	Evernia	25
Everniopsis_trulla	Parmeliaceae	1	Everniopsis	26
Everniopsis_trulla_5706	Parmeliaceae	1	Everniopsis	26
Flavoparmelia_baltimorensis	Parmeliaceae	1	Flavoparmelia	27
Flavoparmelia_caperata2	Parmeliaceae	1	Flavoparmelia	27
Flavoparmelia_haysomii	Parmeliaceae	1	Flavoparmelia	27
Flavoparmelia_marchantii	Parmeliaceae	1	Flavoparmelia	27
Flavoparmelia_soredians2	Parmeliaceae	1	Flavoparmelia	27
Flavoparmelia_springtonensis	Parmeliaceae	1	Flavoparmelia	27
Flavoparmelia_subambigua	Parmeliaceae	1	Flavoparmelia	27
Flavoparmelia_citrinescens	Parmeliaceae	1	Flavoparmelia	28
Flavopunctelia_flaventior	Parmeliaceae	1	Flavopunctelia	29
Flavopunctelia_soredica	Parmeliaceae	1	Flavopunctelia	29
Hypotrachyna_cirrhata	Parmeliaceae	1	Hypotrachyna	31
Hypotrachyna_dubitans	Parmeliaceae	1	Hypotrachyna	31
Hypotrachyna_kaernefeltii	Parmeliaceae	1	Hypotrachyna	31
Hypotrachyna_lipidifera	Parmeliaceae	1	Hypotrachyna	31
Hypotrachyna_nepalensis	Parmeliaceae	1	Hypotrachyna	31
Hypotrachyna_sorocheila	Parmeliaceae	1	Hypotrachyna	31
Hypotrachyna_endochloraMAF10379	Parmeliaceae	1	Hypotrachyna	32
Hypotrachyna_imbricatula	Parmeliaceae	1	Hypotrachyna	32
Hypotrachyna_physcioides1	Parmeliaceae	1	Hypotrachyna	32
Hypotrachyna_neodissecta2	Parmeliaceae	1	Hypotrachyna	33
Hypotrachyna_polydactyla	Parmeliaceae	1	Hypotrachyna	33
Hypotrachyna_pseudosinuosa	Parmeliaceae	1	Hypotrachyna	33
Hypotrachyna_revoluta	Parmeliaceae	1	Hypotrachyna	33
Parmelinopsis_afrorevoluta	Parmeliaceae	1	Hypotrachyna	33
Parmelinopsis_horrescens2	Parmeliaceae	1	Hypotrachyna	33
Parmelinopsis_minarum	Parmeliaceae	1	Hypotrachyna	33
Parmelinopsis_neodamaziana	Parmeliaceae	1	Hypotrachyna	33
Parmelinopsis_subfatiscens	Parmeliaceae	1	Hypotrachyna	33
Hypotrachyna_sinuosa	Parmeliaceae	1	Hypotrachyna	34
Imshaugia_aleurites	Parmeliaceae	1	Imshaugia	35
Letharia_columbiana	Parmeliaceae	1	Letharia	50
Lethariella_cashmeriana	Parmeliaceae	1	Letharia	50
Lethariella_togashii_YO6735	Parmeliaceae	1	Lethariella	51
Melanelixia_albertana	Parmeliaceae	1	Melanelixia	52
Melanelixia_californica1	Parmeliaceae	1	Melanelixia	52
Melanelixia_californica3	Parmeliaceae	1	Melanelixia	52
Melanelixia_glabra1	Parmeliaceae	1	Melanelixia	52
Melanelixia_subargentifera2	Parmeliaceae	1	Melanelixia	52

Melanelixia_villosella2	Parmeliaceae	1	Melanelixia	52
Melanelixia_fuliginosa	Parmeliaceae	1	Melanelixia	53
Melanelixia_subaurifera3	Parmeliaceae	1	Melanelixia	53
Melanelixia_glabratuloides	Parmeliaceae	1	Melanelixia	54
Melanelixia_pilliferella	Parmeliaceae	1	Melanelixia	54
Melanelixia_subglabra	Parmeliaceae	1	Melanelixia	55
Melanohalea_aff_exasperata3	Parmeliaceae	1	Melanohalea	56
Melanohalea_elegantula_SD	Parmeliaceae	1	Melanohalea	56
Melanohalea_exasperata_SD	Parmeliaceae	1	Melanohalea	56
Melanohalea_exasperata2	Parmeliaceae	1	Melanohalea	56
Melanohalea_exasperatula_SD	Parmeliaceae	1	Melanohalea	56
Melanohalea_laciniatula3	Parmeliaceae	1	Melanohalea	56
Melanohalea_laciniatula4	Parmeliaceae	1	Melanohalea	56
Melanohalea_multispora_SD	Parmeliaceae	1	Melanohalea	56
Melanohalea_subolivacea_SD	Parmeliaceae	1	Melanohalea	56
Melanohalea_trabeculata	Parmeliaceae	1	Melanohalea	56
Melanohalea_aff_olivacea	Parmeliaceae	1	Melanohalea	57
Melanohalea_gomukhensis_SD	Parmeliaceae	1	Melanohalea	57
Melanohalea_halei_SD	Parmeliaceae	1	Melanohalea	57
Melanohalea_infumata_SD	Parmeliaceae	1	Melanohalea	57
Melanohalea_olivacea	Parmeliaceae	1	Melanohalea	57
Melanohalea_olivacea_SD	Parmeliaceae	1	Melanohalea	57
Melanohalea_olivaceoides_SD	Parmeliaceae	1	Melanohalea	57
Melanohalea_septentrionalis_SD	Parmeliaceae	1	Melanohalea	57
Melanohalea_subelegantula	Parmeliaceae	1	Melanohalea	57
Melanohalea_ushuaiensis_SD	Parmeliaceae	1	Melanohalea	58
Menegazzia_chrysogaster_TROM_L48003	Parmeliaceae	1	Menegazzia	59
Menegazzia_confusa_HO558312	Parmeliaceae	1	Menegazzia	59
Menegazzia_elongata_HO559273	Parmeliaceae	1	Menegazzia	59
Menegazzia_kawesqarica_TROM_L48031	Parmeliaceae	1	Menegazzia	59
Menegazzia_myriotrema	Parmeliaceae	1	Menegazzia	59
Menegazzia_subbullata_HO559272	Parmeliaceae	1	Menegazzia	59
Menegazzia_subpertusa_TROM_L45455	Parmeliaceae	1	Menegazzia	59
Menegazzia_terebrata_IKT_10003	Parmeliaceae	1	Menegazzia	59
Menegazzia_violascens_TROM_L45316	Parmeliaceae	1	Menegazzia	59
Montanelia_disjuncta	Parmeliaceae	1	Montanelia	62
Montanelia_panniformis1	Parmeliaceae	1	Montanelia	62
Montanelia_panniformis3	Parmeliaceae	1	Montanelia	62
Montanelia_sorediata	Parmeliaceae	1	Montanelia	62
Montanelia_tominii	Parmeliaceae	1	Montanelia	62
Myelochroa_aurulenta	Parmeliaceae	1	Myelochroa	65
Myelochroa_irrugans	Parmeliaceae	1	Myelochroa	65
Myelochroa_metarevoluta	Parmeliaceae	1	Myelochroa	65
Nipponoparmelia_laevior	Parmeliaceae	1	Nipponoparmelia	66
Nipponoparmelia_ricasolioides	Parmeliaceae	1	Nipponoparmelia	66
Nodobryoria_abbreviata_lls01	Parmeliaceae	1	Nodobryoria	67
Notoparmelia_crambidiocarpa	Parmeliaceae	1	Notoparmelia	68
Notoparmelia_cunninghamii	Parmeliaceae	1	Notoparmelia	68
Notoparmelia_signifera	Parmeliaceae	1	Notoparmelia	68
Notoparmelia_subtestacea	Parmeliaceae	1	Notoparmelia	68
Notoparmelia_tenuirima	Parmeliaceae	1	Notoparmelia	68
Omphalodium_pisacomense	Parmeliaceae	1	Omphalodium	69
Oropogon_atranorinus_4036	Parmeliaceae	1	Oropogon	70
Oropogon_evernicus_4032	Parmeliaceae	1	Oropogon	70
Oropogon_fumosus_cr1	Parmeliaceae	1	Oropogon	70
Oropogon_loxensis_cr2	Parmeliaceae	1	Oropogon	70
Oropogon_sp1_cr10	Parmeliaceae	1	Oropogon	70
Oropogon_sperlingii_4072	Parmeliaceae	1	Oropogon	70
Oropogon_striatulus_cr7	Parmeliaceae	1	Oropogon	70

Pannoparmelia_angustata_MWE145	Parmeliaceae	1	Pannoparmelia	71
Pannoparmelia_wilsonii2109	Parmeliaceae	1	Pannoparmelia	71
Parmelia_barrenoae	Parmeliaceae	1	Parmelia	72
Parmelia_discordans	Parmeliaceae	1	Parmelia	72
Parmelia_saxatilis	Parmeliaceae	1	Parmelia	72
Parmelia_serrana	Parmeliaceae	1	Parmelia	72
Parmelia_squarrosa	Parmeliaceae	1	Parmelia	72
Parmelia_sulcata2	Parmeliaceae	1	Parmelia	72
Parmelina_carporrhizans	Parmeliaceae	1	Parmelina	73
Parmelina_pastillifera	Parmeliaceae	1	Parmelina	73
Parmelina_quercina1	Parmeliaceae	1	Parmelina	73
Parmelina_tiliacea	Parmeliaceae	1	Parmelina	73
Parmelinella_wallichiana	Parmeliaceae	1	Parmelinella	74
Parmeliopsis_ambigua	Parmeliaceae	1	Parmeliopsis	75
Parmeliopsis_hyperopta	Parmeliaceae	1	Parmeliopsis	75
Parmotrema_cetratum	Parmeliaceae	1	Parmotrema	76
Parmotrema_crinitum	Parmeliaceae	1	Parmotrema	76
Parmotrema_fistulatum	Parmeliaceae	1	Parmotrema	76
Parmotrema_haitiense	Parmeliaceae	1	Parmotrema	76
Parmotrema_hypoleucinum	Parmeliaceae	1	Parmotrema	76
Parmotrema_norsticticatum	Parmeliaceae	1	Parmotrema	76
Parmotrema_perforatum	Parmeliaceae	1	Parmotrema	76
Parmotrema_perlatum	Parmeliaceae	1	Parmotrema	76
Parmotrema_pilosum	Parmeliaceae	1	Parmotrema	76
Parmotrema_reticulatum1	Parmeliaceae	1	Parmotrema	76
Phacopsis_huuskonenii_HL361S2	Parmeliaceae	1	Phacopsis	77
Protousnea_magellanica_MWE157	Parmeliaceae	1	Protousnea	77
Protousnea_sp_2271	Parmeliaceae	1	Protousnea	77
Protousnea_sp_2274	Parmeliaceae	1	Protousnea	77
Platismatia_glauca	Parmeliaceae	1	Platismatia	78
Platismatia_norvegica	Parmeliaceae	1	Platismatia	78
Platismatia_tuckermanii2229	Parmeliaceae	1	Platismatia	78
Protoparmelia_badiaA_71474_003	Parmeliaceae	1	Protoparmelia	80
Protoparmelia_badiaB1_SD_2	Parmeliaceae	1	Protoparmelia	80
Protoparmelia_badiaC_19437_BA140185	Parmeliaceae	1	Protoparmelia	80
Protoparmelia_hypotremella_14305A_HY318	Parmeliaceae	1	Protoparmelia	80
Protoparmelia_memnonia_9612_ME037	Parmeliaceae	1	Protoparmelia	80
Protoparmelia_montagneiA_19465_MO310	Parmeliaceae	1	Protoparmelia	80
Protoparmelia_montagneiB_19459_MO306	Parmeliaceae	1	Protoparmelia	80
Protoparmelia_montagneiC_19427_MO140175	Parmeliaceae	1	Protoparmelia	80
Protoparmelia_oleagina_10816_OL283	Parmeliaceae	1	Protoparmelia	80
Protoparmelia_picea	Parmeliaceae	1	Protoparmelia	80
Protoparmelia_capitata_55885_CA323	Parmeliaceae	1	Protoparmelia	81
Protoparmelia_corallifera_6984_CO299	Parmeliaceae	1	Protoparmelia	81
Protoparmelia_orientalis_6922_OR296	Parmeliaceae	1	Protoparmelia	84
Protoparmelia_pulchra_37097_PU064	Parmeliaceae	1	Protoparmelia	84
Pseudephebe_pubescens	Parmeliaceae	1	Pseudephebe	85
Pseudoparmelia_cyphellata_8609	Parmeliaceae	1	Pseudoparmelia	86
Pseudoparmelia_floridensisKS3	Parmeliaceae	1	Pseudoparmelia	86
Pseudoparmelia_uleana8706	Parmeliaceae	1	Pseudoparmelia	86
Psiloparmelia_denotata	Parmeliaceae	1	Psiloparmelia	87
Psiloparmelia_sp	Parmeliaceae	1	Psiloparmelia	87
Relicina_subnigra	Parmeliaceae	1	Relicina	93
Relicina_sydneyensis	Parmeliaceae	1	Relicina	93
Relicinopsis_intertexta_1083	Parmeliaceae	1	Relicinopsis	94
Relicinopsis_rahengensis_1084	Parmeliaceae	1	Relicinopsis	94
Relicinopsis_stevensii_1073	Parmeliaceae	1	Relicinopsis	94
Remototrachyna_ciliata	Parmeliaceae	1	Remototrachyna	95
Remototrachyna_costaricensis	Parmeliaceae	1	Remototrachyna	95

Remototrachyna_flexilis	Parmeliaceae	1	Remototrachyna	95
Remototrachyna_incognita1	Parmeliaceae	1	Remototrachyna	95
Remototrachyna_infirma2	Parmeliaceae	1	Remototrachyna	95
Remototrachyna_scytophylla1	Parmeliaceae	1	Remototrachyna	95
Sulcaria_sulcata	Parmeliaceae	1	Sulcaria	101
Sulcaria_virens	Parmeliaceae	1	Sulcaria	101
Usnea_antarctica	Parmeliaceae	1	Usnea	103
Usnea_articulata_ART_02	Parmeliaceae	1	Usnea	103
Usnea_cornuta_cor42FRST	Parmeliaceae	1	Usnea	103
Usnea_filipendula_YO6771	Parmeliaceae	1	Usnea	103
Usnea_florida	Parmeliaceae	1	Usnea	103
Usnea_fragilescens_fra96BO	Parmeliaceae	1	Usnea	103
Usnea_fulvoreagens_LAP_04	Parmeliaceae	1	Usnea	103
Usnea_glabrata_gla113CHS	Parmeliaceae	1	Usnea	103
Usnea_glabrescens_GLA_17	Parmeliaceae	1	Usnea	103
Usnea_hirta_HIR_01	Parmeliaceae	1	Usnea	103
Usnea_mutabilis_YO4407	Parmeliaceae	1	Usnea	103
Usnea_rubicunda	Parmeliaceae	1	Usnea	103
Usnea_sphacelata	Parmeliaceae	1	Usnea	103
Usnea_subaranea123	Parmeliaceae	1	Usnea	103
Usnea_subfloridana	Parmeliaceae	1	Usnea	103
Usnea_trachycarpa	Parmeliaceae	1	Usnea	103
Usnea_wasmuthii_SUB_03	Parmeliaceae	1	Usnea	103
Usnea_pectinata_YO4373	Parmeliaceae	1	Usnea	104
Usnea_trichodeoides_YO5316	Parmeliaceae	1	Usnea	105
Xanthoparmelia_azaiensis	Parmeliaceae	1	Xanthoparmelia	106
Xanthoparmelia_brachinaensis	Parmeliaceae	1	Xanthoparmelia	106
Xanthoparmelia_chlorochroa_536	Parmeliaceae	1	Xanthoparmelia	106
Xanthoparmelia_conspersa	Parmeliaceae	1	Xanthoparmelia	106
Xanthoparmelia_crespoae1	Parmeliaceae	1	Xanthoparmelia	106
Xanthoparmelia_cumberlandia_nybg02	Parmeliaceae	1	Xanthoparmelia	106
Xanthoparmelia_dierythra_6510	Parmeliaceae	1	Xanthoparmelia	106
Xanthoparmelia_exornata	Parmeliaceae	1	Xanthoparmelia	106
Xanthoparmelia_hottentota	Parmeliaceae	1	Xanthoparmelia	106
Xanthoparmelia_isidiovagans	Parmeliaceae	1	Xanthoparmelia	106
Xanthoparmelia_loxodes1	Parmeliaceae	1	Xanthoparmelia	106
Xanthoparmelia_mougeotii2	Parmeliaceae	1	Xanthoparmelia	106
Xanthoparmelia_pokornyii2	Parmeliaceae	1	Xanthoparmelia	106
Xanthoparmelia_protomatrae	Parmeliaceae	1	Xanthoparmelia	106
Xanthoparmelia_saxetii_538	Parmeliaceae	1	Xanthoparmelia	106
Xanthoparmelia_semiviridis	Parmeliaceae	1	Xanthoparmelia	106
Xanthoparmelia_stenophylla	Parmeliaceae	1	Xanthoparmelia	106
Xanthoparmelia_subdiffluens	Parmeliaceae	1	Xanthoparmelia	106
Xanthoparmelia_tinctina1	Parmeliaceae	1	Xanthoparmelia	106
Xanthoparmelia_vicentei1	Parmeliaceae	1	Xanthoparmelia	106
Xanthoparmelia_wyomingica_826	Parmeliaceae	1	Xanthoparmelia	106
Cladia_aggregata	Cladoniaceae	2	Cladia	17
Cladia_dumicola	Cladoniaceae	2	Cladia	17
Cladia_schizopora	Cladoniaceae	2	Cladia	17
Cladonia_caroliniana	Cladoniaceae	2	Cladonia	18
Cladonia_stipitata	Cladoniaceae	2	Cladonia	18
Cladonia_sulcata	Cladoniaceae	2	Cladonia	18
Cladonia_rangiferina	Cladoniaceae	2	Cladina	19
Metus_conglomeratus	Cladoniaceae	2	Metus	60
Pycnothelia_papillaria	Cladoniaceae	2	Pycnothelia	88
Gypsoplaca_macrophylla	Gypsoplacaceae	3	Gyposoplaca	30
Lecanora_achroa0	Lecanoraceae	4	Lecanora	36
Lecanora_flavopallida0	Lecanoraceae	4	Lecanora	38
Lecanora_tropica2	Lecanoraceae	4	Lecanora	42

Lecidella_patavina0	Lecanoraceae	4	Lecidella	47
Lecidella_stigmatea1	Lecanoraceae	4	Lecidella	47
Lecanora_carpinea	Lecanoraceae	5	Lecanorella	37
Lecanora_hybocarpa	Lecanoraceaex	5	Lecanorella	40
Lecanora_paramerae	Lecanoraceaex	5	Lecanorella	40
Lecanora_sulphurea	Lecanoraceaex	5	Lecanorella	41
Lecanora_garovaglii0	Lecanoraceae	6	Protoparmeliopsis	39
Lecanora_muralis2	Lecanoraceae	6	Protoparmeliopsis	39
Rhizoplaca_peltata2	Lecanoraceae	6	Protoparmeliopsis	39
Rhizoplaca_chrysoleuca1	Lecanoraceae	6	Rhizoplaca	96
Rhizoplaca_haydenii	Lecanoraceae	6	Rhizoplaca	97
Rhizoplaca_porterii	Lecanoraceae	6	Rhizoplaca	97
Lecidea_floridensis	Lecideaceaex	7	Lecideaxx	43
Lecidea_sp1	Lecideaceaex	7	Lecideaxx	46
Lecidea_nylanderi	Lecideaceae	8	Lecidea	44
Lecidea_roseotincta	Lecideaceae	8	Lecidea	45
Lepraria_bergensis	Stereocaulaceae	9	Leprariella	48
Lepraria_lobificans	Stereocaulaceae	9	Lepraria	49
Stereocaulon_paschale	Stereocaulaceae	9	Stereocaulon	100
Stereocaulon_tomentosum	Stereocaulaceae	9	Stereocaulon	100
Miriquidica_complanata	Miriquidicaceae	10	Miriquidica	61
Miriquidica_garovagli	Miriquidicaceae	10	Miriquidica	61
Miriquidica_leucophaea	Miriquidicaceae	10	Miriquidica	61
Protoparmelia_atriseda_26046_007	Miriquidicaceae	10	Miriquidica	79
Protoparmelia_cupreobadiaA_8631_028	Miriquidicaceae	10	Miriquidica	79
Protoparmelia_phaeonesos_13365_082	Miriquidicaceae	10	Miriquidica	79
Protoparmelia_leproloma_3046_012	Miriquidicaceae	10	Miriquidica	82
Protoparmelia_nephaea_120032	Miriquidicaceae	10	Miriquidica	83
Mycoblastus_affinis	Tephromelataceae	11	Mycoblastus	63
Mycoblastus_sanguinarius	Tephromelataceae	11	Mycoblastellus	64
Tephromela_atra	Tephromelataceae	11	Tephromela	102
Pyrrhosporea_laeta36817	Ramboldiaceae	12	Ramboldia	89
Pyrrhosporea_sanguinolenta	Ramboldiaceae	12	Ramboldia	90
Ramboldia_sanguinolenta	Ramboldiaceae	12	Ramboldia	90
Ramboldia_brunneocarpa	Ramboldiaceae	12	Ramboldia	91
Ramboldia_startii28664	Ramboldiaceae	12	Ramboldia	92
Squamarina_cartilaginea	Squamarinaceae	13	Squamarina	98
Squamarina_lentigera	Squamarinaceae	13	Squamarina	98
Squamarina_gypsacea	Squamarinaceae	13	Squamarina	99

	OldFam	NewFam	OldGen	NewGen
Ahtiana_pallidula	Parmeliaceae	1	Ahtiana	1
Allocetraria_ambigua	Parmeliaceae	1	Allocetraria	1
Allocetraria_flavonigrescens	Parmeliaceae	1	Allocetraria	1
Allocetraria_globulans	Parmeliaceae	1	Allocetraria	1
Allocetraria_stracheyi_AST_02	Parmeliaceae	1	Allocetraria	1
Arctocetraria_andrejewii	Parmeliaceae	1	Arctocetraria	1
Arctocetraria_nigricascens	Parmeliaceae	1	Arctocetraria	1
Cetraria_islandica	Parmeliaceae	1	Cetraria	1
Cetraria_laevigata	Parmeliaceae	1	Cetraria	1
Cetraria_muricata	Parmeliaceae	1	Cetraria	1
Cetraria_nigricans	Parmeliaceae	1	Cetraria	1
Cetraria_odontella	Parmeliaceae	1	Cetraria	1
Cetraria_sepincola_CSE_01	Parmeliaceae	1	Cetraria	1
Cetrariella_delisei	Parmeliaceae	1	Cetrariella	1
Cetrariella_fastigiata	Parmeliaceae	1	Cetrariella	1
Cetrariella_fastigiata_CFA_02	Parmeliaceae	1	Cetrariella	1
Cetreliopsis_rhytidocarpa	Parmeliaceae	1	Cetreliopsis	1
Flavocetraria_cucullata	Parmeliaceae	1	Flavocetraria	1
Flavocetraria_nivalis	Parmeliaceae	1	Flavocetrariella	1
Kaernefeltia_merrillii	Parmeliaceae	1	Kaernefeltia	1
Masonhalea_inermis_551a	Parmeliaceae	1	Masonhalea	1
Masonhalea_richardsonii	Parmeliaceae	1	Masonhalea	1
Melanelia_commixta	Parmeliaceae	1	Melanelicetraria	1
Melanelia_sorediella	Parmeliaceae	1	Melanelicetraria	1
Melanelia_culbersonii_3992	Parmeliaceae	1	Melaneliculbersonia	1
Cetraria_obtusata	Parmeliaceae	1	Neocetraria	1
Nephromopsis_komarovii	Parmeliaceae	1	Nephromopsis	1
Nephromopsis_laureri	Parmeliaceae	1	Nephromopsis	1
Nephromopsis_leucostigma	Parmeliaceae	1	Nephromopsis	1
Nephromopsis_nephromoides	Parmeliaceae	1	Nephromopsis	1
Nephromopsis_pallescens	Parmeliaceae	1	Nephromopsis	1
Tuckermanella_coralligera	Parmeliaceae	1	Tuckermanella	1
Tuckermanella_fendleri	Parmeliaceae	1	Tuckermanella	1
Tuckermannopsis_chlorophylla	Parmeliaceae	1	Tuckermannopsis	1
Tuckermannopsis_ciliaris	Parmeliaceae	1	Tuckermannopsis	1
Tuckermannopsis_orbata	Parmeliaceae	1	Tuckermannopsis	1
Usnocetraria_oakesiana	Parmeliaceae	1	Usnocetraria	1
Vulpicida_canadensis_CAN_15	Parmeliaceae	1	Vulpicida	1
Vulpicida_juniperinus_JUN_07	Parmeliaceae	1	Vulpicida	1
Vulpicida_pinastri	Parmeliaceae	1	Vulpicida	1
Vulpicida_tubulosus_JUN_14	Parmeliaceae	1	Vulpicida	1
Vulpicida_viridis_VIR_10	Parmeliaceae	1	Vulpicida	1
Alectoria_arctica_S146	Parmeliaceae	1	Alectoria	2
Alectoria_nigricans	Parmeliaceae	1	Alectoria	2
Alectoria_ochroleuca	Parmeliaceae	1	Alectoria	2
Alectoria_sarmentosa	Parmeliaceae	1	Alectoria	2
Allantoparmelia_almquistii_5158	Parmeliaceae	1	Allantoparmelia	3
Allantoparmelia_alpicola	Parmeliaceae	1	Allantoparmelia	3
Anzia_colpodes	Parmeliaceae	1	Anzia	4
Anzia_flavotenuis	Parmeliaceae	1	Anzia	5
Anzia_mahaeliyensis	Parmeliaceae	1	Anzia	5
Arctoparmelia_centrifuga	Parmeliaceae	1	Arctoparmelia	6
Arctoparmelia_incurva_MWE10	Parmeliaceae	1	Arctoparmelia	6
Austroparmelina_endoleuca2	Parmeliaceae	1	Austroparmelina	7
Austroparmelina_macrospora	Parmeliaceae	1	Austroparmelina	7
Austroparmelina_pruinata	Parmeliaceae	1	Austroparmelina	7
Austroparmelina_pseudorelicina	Parmeliaceae	1	Austroparmelina	7
Brodoa_atrofusca	Parmeliaceae	1	Brodoa	8

Brodoa_intestiniformis	Parmeliaceae	1	Brodoa	8
Brodoa_oroarctica	Parmeliaceae	1	Brodoa	8
Hypogymnia_bitteri30678	Parmeliaceae	1	Hypogymnia	8
Hypogymnia_hultenii2	Parmeliaceae	1	Hypogymnia	8
Hypogymnia_imshaugii1	Parmeliaceae	1	Hypogymnia	8
Hypogymnia_imshaugii2	Parmeliaceae	1	Hypogymnia	8
Hypogymnia_lophyrea2	Parmeliaceae	1	Hypogymnia	8
Hypogymnia_lugubris3014	Parmeliaceae	1	Hypogymnia	8
Hypogymnia_mollisD79	Parmeliaceae	1	Hypogymnia	8
Hypogymnia_mundata3013	Parmeliaceae	1	Hypogymnia	8
Hypogymnia_physodes	Parmeliaceae	1	Hypogymnia	8
Hypogymnia_pulverata_2277	Parmeliaceae	1	Hypogymnia	8
Hypogymnia_pulverata_2281	Parmeliaceae	1	Hypogymnia	8
Hypogymnia_rugosa3822	Parmeliaceae	1	Hypogymnia	8
Hypogymnia_subphysodes_2280	Parmeliaceae	1	Hypogymnia	8
Hypogymnia_tasmanica3012	Parmeliaceae	1	Hypogymnia	8
Hypogymnia_vittata	Parmeliaceae	1	Hypogymnia	8
Pseudevernia_consocians	Parmeliaceae	1	Pseudevernia	8
Pseudevernia_furfuracea	Parmeliaceae	1	Pseudevernia	8
Bryocaulon_divergens_MWE158	Parmeliaceae	1	Bryocaulon	9
Bryocaulon_pseudosatoanum_YO8239	Parmeliaceae	1	Bryocaulon	9
Bryocaulon_satoanumMWE163	Parmeliaceae	1	Bryocaulon	9
Bryoria_americana_S329	Parmeliaceae	1	Bryoria	10
Bryoria_bicolor_L156	Parmeliaceae	1	Bryoria	10
Bryoria_capillaris	Parmeliaceae	1	Bryoria	10
Bryoria_fremontii	Parmeliaceae	1	Bryoria	10
Bryoria_furcellata_L147	Parmeliaceae	1	Bryoria	10
Bryoria_fuscescens_S56	Parmeliaceae	1	Bryoria	10
Bryoria_glabra_L186	Parmeliaceae	1	Bryoria	10
Bryoria_implexa_L244a	Parmeliaceae	1	Bryoria	10
Bryoria_nadvornikiana_S79	Parmeliaceae	1	Bryoria	10
Bryoria_simplicior_S30b	Parmeliaceae	1	Bryoria	10
Bryoria_smithii_S65	Parmeliaceae	1	Bryoria	10
Bryoria_trichodes	Parmeliaceae	1	Bryoria	10
Bulbothrix_apophysata	Parmeliaceae	1	Bulbothricella	11
Bulbothrix_coronata	Parmeliaceae	1	Bulbothricella	11
Bulbothrix_decurtata	Parmeliaceae	1	Bulbothrix	12
Bulbothrix_isidiza1	Parmeliaceae	1	Bulbothrix	12
Bulbothrix_isidiza2	Parmeliaceae	1	Bulbothrix	12
Bulbothrix_meizospora	Parmeliaceae	1	Bulbothrix	12
Bulbothrix_meizospora2	Parmeliaceae	1	Bulbothrix	12
Bulbothrix_sensibilis	Parmeliaceae	1	Bulbothrix	12
Bulbothrix_setschwanensis	Parmeliaceae	1	Bulbothrix	12
Bulbothrix_tabacina	Parmeliaceae	1	Bulbothrix	12
Canoparmelia_carneopruinata	Parmeliaceae	1	Crespoa	13
Nesolechia_oxyspora_16840	Parmeliaceae	1	Nesolechia	13
Punctelia_aff_bolliana	Parmeliaceae	1	Punctelia	13
Punctelia_borreri2	Parmeliaceae	1	Punctelia	13
Punctelia_pseudocoralloidea	Parmeliaceae	1	Punctelia	13
Punctelia_reddenda	Parmeliaceae	1	Punctelia	13
Punctelia_rudecta	Parmeliaceae	1	Punctelia	13
Punctelia_subflava	Parmeliaceae	1	Punctelia	13
Punctelia_subrudecta3	Parmeliaceae	1	Punctelia	13
Punctelia_ullophylla	Parmeliaceae	1	Punctelia	13
Canoparmelia_caroliniana	Parmeliaceae	1	Canoparmelia	14
Canoparmelia_nairobiensis	Parmeliaceae	1	Canoparmelia	14
Canoparmelia_inhaminensis	Parmeliaceae	1	Crespoa	14
Canoparmelia_schelpei	Parmeliaceae	1	Crespoa	14
Canoparmelia_concrescens	Parmeliaceae	1	Canoparmelia	15

Canoparmelia_texana	Parmeliaceae	1	Canoparmelia	15
Canoparmelia_crozalsiana	Parmeliaceae	1	Crespoa	15
Canoparmelia_Punctelia_sp	Parmeliaceae	1	Newgenus	15
Cetrelia_cetrarioides	Parmeliaceae	1	Cetrelia	16
Cetrelia_olivetorum	Parmeliaceae	1	Cetrelia	16
Cetrelia_pseudolivetorum	Parmeliaceae	1	Cetrelia	16
Coelopogon_abraxus_4253	Parmeliaceae	1	Coelopogon	20
Coelopogon_epiphorellus_4254	Parmeliaceae	1	Coelopogon	20
Coelopogon_epiphorellus_MWE156	Parmeliaceae	1	Coelopogon	20
Cornicularia_normoerica	Parmeliaceae	1	Cornicularia	21
Dactylina_arctica_4855	Parmeliaceae	1	Dactylina	22
Dactylina_ramulosa_4902	Parmeliaceae	1	Dactylina	22
Emodomelanelia_masonii	Parmeliaceae	1	Emodomelanelia	23
Esslingeriana_idahoensis_4823	Parmeliaceae	1	Esslingeriana	24
Melanelia_hepatizon	Parmeliaceae	1	Melanelia	24
Melanelia_stygia	Parmeliaceae	1	Melanelia	24
Evernia_divaricata	Parmeliaceae	1	Evernia	25
Evernia_mesomorpha	Parmeliaceae	1	Evernia	25
Evernia_prunastri	Parmeliaceae	1	Evernia	25
Everniopsis_trulla	Parmeliaceae	1	Everniopsis	26
Everniopsis_trulla_5706	Parmeliaceae	1	Everniopsis	26
Flavoparmelia_baltimorensis	Parmeliaceae	1	Flavoparmelia	27
Flavoparmelia_caperata2	Parmeliaceae	1	Flavoparmelia	27
Flavoparmelia_haysomii	Parmeliaceae	1	Flavoparmelia	27
Flavoparmelia_marchantii	Parmeliaceae	1	Flavoparmelia	27
Flavoparmelia_soredians2	Parmeliaceae	1	Flavoparmelia	27
Flavoparmelia_springtonensis	Parmeliaceae	1	Flavoparmelia	27
Flavoparmelia_subambigua	Parmeliaceae	1	Flavoparmelia	27
Flavoparmelia_citrinescens	Parmeliaceae	1	Flavoparmelia	28
Flavopunctelia_flaventior	Parmeliaceae	1	Flavopunctelia	29
Flavopunctelia_soredica	Parmeliaceae	1	Flavopunctelia	29
Hypotrachyna_cirrhata	Parmeliaceae	1	Hypotrachyna	31
Hypotrachyna_dubitans	Parmeliaceae	1	Hypotrachyna	31
Hypotrachyna_kaernefeltii	Parmeliaceae	1	Hypotrachyna	31
Hypotrachyna_lipidifera	Parmeliaceae	1	Hypotrachyna	31
Hypotrachyna_nepalensis	Parmeliaceae	1	Hypotrachyna	31
Hypotrachyna_sorocheila	Parmeliaceae	1	Hypotrachyna	31
Hypotrachyna_endochloraMAF10379	Parmeliaceae	1	Hypotrachyna	32
Hypotrachyna_imbricatula	Parmeliaceae	1	Hypotrachyna	32
Hypotrachyna_physcioides1	Parmeliaceae	1	Hypotrachyna	32
Hypotrachyna_neodissecta2	Parmeliaceae	1	Hypotrachyna	33
Hypotrachyna_polydactyla	Parmeliaceae	1	Hypotrachyna	33
Hypotrachyna_pseudosinuosa	Parmeliaceae	1	Hypotrachyna	33
Hypotrachyna_revoluta	Parmeliaceae	1	Hypotrachyna	33
Parmelinopsis_afrorevoluta	Parmeliaceae	1	Hypotrachyna	33
Parmelinopsis_horrescens2	Parmeliaceae	1	Hypotrachyna	33
Parmelinopsis_minarum	Parmeliaceae	1	Hypotrachyna	33
Parmelinopsis_neodamaziana	Parmeliaceae	1	Hypotrachyna	33
Parmelinopsis_subfatiscens	Parmeliaceae	1	Hypotrachyna	33
Hypotrachyna_sinuosa	Parmeliaceae	1	Hypotrachyna	34
Imshaugia_aleurites	Parmeliaceae	1	Imshaugia	35
Letharia_columbiana	Parmeliaceae	1	Letharia	50
Lethariella_cashmeriana	Parmeliaceae	1	Letharia	50
Lethariella_togashii_YO6735	Parmeliaceae	1	Lethariella	51
Melanelixia_albertana	Parmeliaceae	1	Melanelixia	52
Melanelixia_californica1	Parmeliaceae	1	Melanelixia	52
Melanelixia_californica3	Parmeliaceae	1	Melanelixia	52
Melanelixia_glabra1	Parmeliaceae	1	Melanelixia	52
Melanelixia_subargentifera2	Parmeliaceae	1	Melanelixia	52

Melanelixia_villosella2	Parmeliaceae	1	Melanelixia	52
Melanelixia_fuliginosa	Parmeliaceae	1	Melanelixia	53
Melanelixia_subaurifera3	Parmeliaceae	1	Melanelixia	53
Melanelixia_glabratuloides	Parmeliaceae	1	Melanelixia	54
Melanelixia_pilliferella	Parmeliaceae	1	Melanelixia	54
Melanelixia_subglabra	Parmeliaceae	1	Melanelixia	55
Melanohalea_aff_exasperata3	Parmeliaceae	1	Melanohalea	56
Melanohalea_elegantula_SD	Parmeliaceae	1	Melanohalea	56
Melanohalea_exasperata_SD	Parmeliaceae	1	Melanohalea	56
Melanohalea_exasperata2	Parmeliaceae	1	Melanohalea	56
Melanohalea_exasperatula_SD	Parmeliaceae	1	Melanohalea	56
Melanohalea_laciniatula3	Parmeliaceae	1	Melanohalea	56
Melanohalea_laciniatula4	Parmeliaceae	1	Melanohalea	56
Melanohalea_multispora_SD	Parmeliaceae	1	Melanohalea	56
Melanohalea_subolivacea_SD	Parmeliaceae	1	Melanohalea	56
Melanohalea_trabeculata	Parmeliaceae	1	Melanohalea	56
Melanohalea_aff_olivacea	Parmeliaceae	1	Melanohalea	57
Melanohalea_gomukhensis_SD	Parmeliaceae	1	Melanohalea	57
Melanohalea_halei_SD	Parmeliaceae	1	Melanohalea	57
Melanohalea_infumata_SD	Parmeliaceae	1	Melanohalea	57
Melanohalea_olivacea	Parmeliaceae	1	Melanohalea	57
Melanohalea_olivacea_SD	Parmeliaceae	1	Melanohalea	57
Melanohalea_olivaceoides_SD	Parmeliaceae	1	Melanohalea	57
Melanohalea_septentrionalis_SD	Parmeliaceae	1	Melanohalea	57
Melanohalea_subelegantula	Parmeliaceae	1	Melanohalea	57
Melanohalea_ushuaiensis_SD	Parmeliaceae	1	Melanohalea	58
Menegazzia_chrysogaster_TROM_L48003	Parmeliaceae	1	Menegazzia	59
Menegazzia_confusa_HO558312	Parmeliaceae	1	Menegazzia	59
Menegazzia_elongata_HO559273	Parmeliaceae	1	Menegazzia	59
Menegazzia_kawesqarica_TROM_L48031	Parmeliaceae	1	Menegazzia	59
Menegazzia_myriotrema	Parmeliaceae	1	Menegazzia	59
Menegazzia_subbullata_HO559272	Parmeliaceae	1	Menegazzia	59
Menegazzia_subpertusa_TROM_L45455	Parmeliaceae	1	Menegazzia	59
Menegazzia_terebrata_IKT_10003	Parmeliaceae	1	Menegazzia	59
Menegazzia_violascens_TROM_L45316	Parmeliaceae	1	Menegazzia	59
Montanelia_disjuncta	Parmeliaceae	1	Montanelia	62
Montanelia_panniformis1	Parmeliaceae	1	Montanelia	62
Montanelia_panniformis3	Parmeliaceae	1	Montanelia	62
Montanelia_sorediata	Parmeliaceae	1	Montanelia	62
Montanelia_tominii	Parmeliaceae	1	Montanelia	62
Myelochroa_aurulenta	Parmeliaceae	1	Myelochroa	65
Myelochroa_irrugans	Parmeliaceae	1	Myelochroa	65
Myelochroa_metarevoluta	Parmeliaceae	1	Myelochroa	65
Nipponoparmelia_laevior	Parmeliaceae	1	Nipponoparmelia	66
Nipponoparmelia_ricasolioides	Parmeliaceae	1	Nipponoparmelia	66
Nodobryoria_abbreviata_lls01	Parmeliaceae	1	Nodobryoria	67
Notoparmelia_crambidiocarpa	Parmeliaceae	1	Notoparmelia	68
Notoparmelia_cunninghamii	Parmeliaceae	1	Notoparmelia	68
Notoparmelia_signifera	Parmeliaceae	1	Notoparmelia	68
Notoparmelia_subtestacea	Parmeliaceae	1	Notoparmelia	68
Notoparmelia_tenuirima	Parmeliaceae	1	Notoparmelia	68
Omphalodium_pisacomense	Parmeliaceae	1	Omphalodium	69
Oropogon_atranorinus_4036	Parmeliaceae	1	Oropogon	70
Oropogon_evernicus_4032	Parmeliaceae	1	Oropogon	70
Oropogon_fumosus_cr1	Parmeliaceae	1	Oropogon	70
Oropogon_loxensis_cr2	Parmeliaceae	1	Oropogon	70
Oropogon_sp1_cr10	Parmeliaceae	1	Oropogon	70
Oropogon_sperlingii_4072	Parmeliaceae	1	Oropogon	70
Oropogon_striatulus_cr7	Parmeliaceae	1	Oropogon	70

Pannoparmelia_angustata_MWE145	Parmeliaceae	1	Pannoparmelia	71
Pannoparmelia_wilsonii2109	Parmeliaceae	1	Pannoparmelia	71
Parmelia_barrenoae	Parmeliaceae	1	Parmelia	72
Parmelia_discordans	Parmeliaceae	1	Parmelia	72
Parmelia_saxatilis	Parmeliaceae	1	Parmelia	72
Parmelia_serrana	Parmeliaceae	1	Parmelia	72
Parmelia_squarrosa	Parmeliaceae	1	Parmelia	72
Parmelia_sulcata2	Parmeliaceae	1	Parmelia	72
Parmelina_carporrhizans	Parmeliaceae	1	Parmelina	73
Parmelina_pastillifera	Parmeliaceae	1	Parmelina	73
Parmelina_quercina1	Parmeliaceae	1	Parmelina	73
Parmelina_tiliacea	Parmeliaceae	1	Parmelina	73
Parmelinella_wallichiana	Parmeliaceae	1	Parmelinella	74
Parmeliopsis_ambigua	Parmeliaceae	1	Parmeliopsis	75
Parmeliopsis_hyperopta	Parmeliaceae	1	Parmeliopsis	75
Parmotrema_cetratum	Parmeliaceae	1	Parmotrema	76
Parmotrema_crinitum	Parmeliaceae	1	Parmotrema	76
Parmotrema_fistulatum	Parmeliaceae	1	Parmotrema	76
Parmotrema_haitiense	Parmeliaceae	1	Parmotrema	76
Parmotrema_hypoleucinum	Parmeliaceae	1	Parmotrema	76
Parmotrema_norsticticatum	Parmeliaceae	1	Parmotrema	76
Parmotrema_perforatum	Parmeliaceae	1	Parmotrema	76
Parmotrema_perlatum	Parmeliaceae	1	Parmotrema	76
Parmotrema_pilosum	Parmeliaceae	1	Parmotrema	76
Parmotrema_reticulatum1	Parmeliaceae	1	Parmotrema	76
Phacopsis_huuskonenii_HL361S2	Parmeliaceae	1	Phacopsis	77
Protousnea_magellanica_MWE157	Parmeliaceae	1	Protousnea	77
Protousnea_sp_2271	Parmeliaceae	1	Protousnea	77
Protousnea_sp_2274	Parmeliaceae	1	Protousnea	77
Platismatia_glauca	Parmeliaceae	1	Platismatia	78
Platismatia_norvegica	Parmeliaceae	1	Platismatia	78
Platismatia_tuckermanii2229	Parmeliaceae	1	Platismatia	78
Protoparmelia_badiaA_71474_003	Parmeliaceae	1	Protoparmelia	80
Protoparmelia_badiaB1_SD_2	Parmeliaceae	1	Protoparmelia	80
Protoparmelia_badiaC_19437_BA140185	Parmeliaceae	1	Protoparmelia	80
Protoparmelia_hypotremella_14305A_HY318	Parmeliaceae	1	Protoparmelia	80
Protoparmelia_memnonia_9612_ME037	Parmeliaceae	1	Protoparmelia	80
Protoparmelia_montagneiA_19465_MO310	Parmeliaceae	1	Protoparmelia	80
Protoparmelia_montagneiB_19459_MO306	Parmeliaceae	1	Protoparmelia	80
Protoparmelia_montagneiC_19427_MO140175	Parmeliaceae	1	Protoparmelia	80
Protoparmelia_oleagina_10816_OL283	Parmeliaceae	1	Protoparmelia	80
Protoparmelia_picea	Parmeliaceae	1	Protoparmelia	80
Protoparmelia_capitata_55885_CA323	Parmeliaceae	1	Protoparmelia	81
Protoparmelia_corallifera_6984_CO299	Parmeliaceae	1	Protoparmelia	81
Protoparmelia_orientalis_6922_OR296	Parmeliaceae	1	Protoparmelia	84
Protoparmelia_pulchra_37097_PU064	Parmeliaceae	1	Protoparmelia	84
Pseudephebe_pubescens	Parmeliaceae	1	Pseudephebe	85
Pseudoparmelia_cyphellata_8609	Parmeliaceae	1	Pseudoparmelia	86
Pseudoparmelia_floridensisKS3	Parmeliaceae	1	Pseudoparmelia	86
Pseudoparmelia_uleana8706	Parmeliaceae	1	Pseudoparmelia	86
Psiloparmelia_denotata	Parmeliaceae	1	Psiloparmelia	87
Psiloparmelia_sp	Parmeliaceae	1	Psiloparmelia	87
Relicina_subnigra	Parmeliaceae	1	Relicina	93
Relicina_sydneyensis	Parmeliaceae	1	Relicina	93
Relicinopsis_intertexta_1083	Parmeliaceae	1	Relicinopsis	94
Relicinopsis_rahengensis_1084	Parmeliaceae	1	Relicinopsis	94
Relicinopsis_stevensii_1073	Parmeliaceae	1	Relicinopsis	94
Remototrachyna_ciliata	Parmeliaceae	1	Remototrachyna	95
Remototrachyna_costaricensis	Parmeliaceae	1	Remototrachyna	95

Remototrachyna_flexilis	Parmeliaceae	1	Remototrachyna	95
Remototrachyna_incognita1	Parmeliaceae	1	Remototrachyna	95
Remototrachyna_infirma2	Parmeliaceae	1	Remototrachyna	95
Remototrachyna_scytophylla1	Parmeliaceae	1	Remototrachyna	95
Sulcaria_sulcata	Parmeliaceae	1	Sulcaria	101
Sulcaria_virens	Parmeliaceae	1	Sulcaria	101
Usnea_antarctica	Parmeliaceae	1	Usnea	103
Usnea_articulata_ART_02	Parmeliaceae	1	Usnea	103
Usnea_cornuta_cor42FRST	Parmeliaceae	1	Usnea	103
Usnea_filipendula_YO6771	Parmeliaceae	1	Usnea	103
Usnea_florida	Parmeliaceae	1	Usnea	103
Usnea_fragilescens_fra96BO	Parmeliaceae	1	Usnea	103
Usnea_fulvoreagens_LAP_04	Parmeliaceae	1	Usnea	103
Usnea_glabrata_gla113CHS	Parmeliaceae	1	Usnea	103
Usnea_glabrescens_GLA_17	Parmeliaceae	1	Usnea	103
Usnea_hirta_HIR_01	Parmeliaceae	1	Usnea	103
Usnea_mutabilis_YO4407	Parmeliaceae	1	Usnea	103
Usnea_rubicunda	Parmeliaceae	1	Usnea	103
Usnea_sphacelata	Parmeliaceae	1	Usnea	103
Usnea_subaranea123	Parmeliaceae	1	Usnea	103
Usnea_subfloridana	Parmeliaceae	1	Usnea	103
Usnea_trachycarpa	Parmeliaceae	1	Usnea	103
Usnea_wasmuthii_SUB_03	Parmeliaceae	1	Usnea	103
Usnea_pectinata_YO4373	Parmeliaceae	1	Usnea	104
Usnea_trichodeoides_YO5316	Parmeliaceae	1	Usnea	105
Xanthoparmelia_azaiensis	Parmeliaceae	1	Xanthoparmelia	106
Xanthoparmelia_brachinaensis	Parmeliaceae	1	Xanthoparmelia	106
Xanthoparmelia_chlorochroa_536	Parmeliaceae	1	Xanthoparmelia	106
Xanthoparmelia_conspersa	Parmeliaceae	1	Xanthoparmelia	106
Xanthoparmelia_crespoae1	Parmeliaceae	1	Xanthoparmelia	106
Xanthoparmelia_cumberlandia_nybg02	Parmeliaceae	1	Xanthoparmelia	106
Xanthoparmelia_dierythra_6510	Parmeliaceae	1	Xanthoparmelia	106
Xanthoparmelia_exornata	Parmeliaceae	1	Xanthoparmelia	106
Xanthoparmelia_hottentota	Parmeliaceae	1	Xanthoparmelia	106
Xanthoparmelia_isidiovagans	Parmeliaceae	1	Xanthoparmelia	106
Xanthoparmelia_loxodes1	Parmeliaceae	1	Xanthoparmelia	106
Xanthoparmelia_mougeotii2	Parmeliaceae	1	Xanthoparmelia	106
Xanthoparmelia_pokornyii2	Parmeliaceae	1	Xanthoparmelia	106
Xanthoparmelia_protomatrae	Parmeliaceae	1	Xanthoparmelia	106
Xanthoparmelia_saxetii_538	Parmeliaceae	1	Xanthoparmelia	106
Xanthoparmelia_semiviridis	Parmeliaceae	1	Xanthoparmelia	106
Xanthoparmelia_stenophylla	Parmeliaceae	1	Xanthoparmelia	106
Xanthoparmelia_subdiffluens	Parmeliaceae	1	Xanthoparmelia	106
Xanthoparmelia_tinctina1	Parmeliaceae	1	Xanthoparmelia	106
Xanthoparmelia_vicentei1	Parmeliaceae	1	Xanthoparmelia	106
Xanthoparmelia_wyomingica_826	Parmeliaceae	1	Xanthoparmelia	106
Cladia_aggregata	Cladoniaceae	2	Cladia	17
Cladia_dumicola	Cladoniaceae	2	Cladia	17
Cladia_schizopora	Cladoniaceae	2	Cladia	17
Cladonia_caroliniana	Cladoniaceae	2	Cladonia	18
Cladonia_stipitata	Cladoniaceae	2	Cladonia	18
Cladonia_sulcata	Cladoniaceae	2	Cladonia	18
Cladonia_rangiferina	Cladoniaceae	2	Cladina	19
Metus_conglomeratus	Cladoniaceae	2	Metus	60
Pycnothelia_papillaria	Cladoniaceae	2	Pycnothelia	88
Gypsoplaca_macrophylla	Gypsoplacaceae	3	Gyposoplaca	30
Lecanora_achroa0	Lecanoraceae	4	Lecanora	36
Lecanora_flavopallida0	Lecanoraceae	4	Lecanora	38
Lecanora_tropica2	Lecanoraceae	4	Lecanora	42

Lecidella_patavina0	Lecanoraceae	4	Lecidella	47
Lecidella_stigmatea1	Lecanoraceae	4	Lecidella	47
Lecanora_carpinea	Lecanoraceae	5	Lecanorella	37
Lecanora_hybocarpa	Lecanoraceaex	5	Lecanorella	40
Lecanora_paramerae	Lecanoraceaex	5	Lecanorella	40
Lecanora_sulphurea	Lecanoraceaex	5	Lecanorella	41
Lecanora_garovaglii0	Lecanoraceae	6	Protoparmeliopsis	39
Lecanora_muralis2	Lecanoraceae	6	Protoparmeliopsis	39
Rhizoplaca_peltata2	Lecanoraceae	6	Protoparmeliopsis	39
Rhizoplaca_chrysoleuca1	Lecanoraceae	6	Rhizoplaca	96
Rhizoplaca_haydenii	Lecanoraceae	6	Rhizoplaca	97
Rhizoplaca_porterii	Lecanoraceae	6	Rhizoplaca	97
Lecidea_floridensis	Lecideaceaex	7	Lecideaxx	43
Lecidea_sp1	Lecideaceaex	7	Lecideaxx	46
Lecidea_nylanderi	Lecideaceae	8	Lecidea	44
Lecidea_roseotincta	Lecideaceae	8	Lecidea	45
Lepraria_bergensis	Stereocaulaceae	9	Leprariella	48
Lepraria_lobificans	Stereocaulaceae	9	Lepraria	49
Stereocaulon_paschale	Stereocaulaceae	9	Stereocaulon	100
Stereocaulon_tomentosum	Stereocaulaceae	9	Stereocaulon	100
Miriquidica_complanata	Miriquidicaceae	10	Miriquidica	61
Miriquidica_garovagli	Miriquidicaceae	10	Miriquidica	61
Miriquidica_leucophaea	Miriquidicaceae	10	Miriquidica	61
Protoparmelia_atriseda_26046_007	Miriquidicaceae	10	Miriquidica	79
Protoparmelia_cupreobadiaA_8631_028	Miriquidicaceae	10	Miriquidica	79
Protoparmelia_phaeonesos_13365_082	Miriquidicaceae	10	Miriquidica	79
Protoparmelia_leproloma_3046_012	Miriquidicaceae	10	Miriquidica	82
Protoparmelia_nephaea_120032	Miriquidicaceae	10	Miriquidica	83
Mycoblastus_affinis	Tephromelataceae	11	Mycoblastus	63
Mycoblastus_sanguinarius	Tephromelataceae	11	Mycoblastellus	64
Tephromela_atra	Tephromelataceae	11	Tephromela	102
Pyrrhosporea_laeta36817	Ramboldiaceae	12	Ramboldia	89
Pyrrhosporea_sanguinolenta	Ramboldiaceae	12	Ramboldia	90
Ramboldia_sanguinolenta	Ramboldiaceae	12	Ramboldia	90
Ramboldia_brunneocarpa	Ramboldiaceae	12	Ramboldia	91
Ramboldia_startii28664	Ramboldiaceae	12	Ramboldia	92
Squamarina_cartilaginea	Squamarinaceae	13	Squamarina	98
Squamarina_lentigera	Squamarinaceae	13	Squamarina	98
Squamarina_gypsacea	Squamarinaceae	13	Squamarina	99
